# Distance-dependent consensus thresholds for generating group-representative structural brain networks

**DOI:** 10.1162/netn_a_00075

**Published:** 2019-03-01

**Authors:** Richard F. Betzel, Alessandra Griffa, Patric Hagmann, Bratislav Mišić

**Affiliations:** Department of Psychological and Brain Sciences, Indiana University, Bloomington, IN, USA; Cognitive Science Program, Indiana University, Bloomington, IN, USA; Program in Neuroscience, Indiana University, Bloomington, IN, USA; Network Science Institute, Indiana University, Bloomington, IN, USA; Dutch Connectome Lab, Department of Complex Trait Genetics, Center for Neurogenomics and Cognitive Research, Amsterdam Neuroscience, VU University, Amsterdam, The Netherlands; Lausanne University Hospital and University of Lausanne (CHUV-UNIL), Lausanne, Switzerland; Montréal Neurological Institute, McGill University, Montréal, Quebec, Canada

**Keywords:** Complex networks, Wiring cost, Connectome, Group-representative, Consensus

## Abstract

Large-scale structural brain networks encode white matter connectivity patterns among distributed brain areas. These connection patterns are believed to support cognitive processes and, when compromised, can lead to neurocognitive deficits and maladaptive behavior. A powerful approach for studying the organizing principles of brain networks is to construct group-representative networks from multisubject cohorts. Doing so amplifies signal to noise ratios and provides a clearer picture of brain network organization. Here, we show that current approaches for generating sparse group-representative networks overestimate the proportion of short-range connections present in a network and, as a result, fail to match subject-level networks along a wide range of network statistics. We present an alternative approach that preserves the connection-length distribution of individual subjects. We have used this method in previous papers to generate group-representative networks, though to date its performance has not been appropriately benchmarked and compared against other methods. As a result of this simple modification, the networks generated using this approach successfully recapitulate subject-level properties, outperforming similar approaches by better preserving features that promote integrative brain function rather than segregative. The method developed here holds promise for future studies investigating basic organizational principles and features of large-scale structural brain networks.

## Author Summary

Sparse structural connectivity data from many subjects can be succinctly represented using appropriate averaging procedures. We show, however, that several popular procedures for doing so generate group-averaged networks with statistics that are dissimilar from the subject-level networks they are intended to represent. These dissimilarities, we argue, arise from the over- and underexpression of short-range and long-distance connections, respectively, in the group-averaged matrix. We present a distance-dependent thresholding procedure that preserves connection length distributions and consequently better matches subject-level networks and their statistics. These findings inform data-driven exploratory analyses of connectomes.

## INTRODUCTION

The human brain is a network composed of neural elements—neurons, populations, and areas—interconnected to one another via synapses, axonal projections, and myelinated fiber tracts, depending on the scale considered (Sporns, Tononi, & Kötter, 2005). These connections shape neural elements’ patterns of input and output and play an important role in determining any given element’s functional properties (Passingham, Stephan, & Kötter, 2002). By modeling neural elements and their connections as the nodes and edges of a graph, we can quantify with summary statistcs the network organization of brains and shed light on their function in health, disease, and development (Sporns, Tononi, & Edelman, 2000).

Though considerable effort has been expended to better understand how different aspects of brain network architecture vary across individuals (Yeh et al., 2016) and covary with behavioral and clinical traits (Gollo et al., 2018; Mišić & Sporns, 2016), studying group-representative brain networks has also proven profitable for understanding the network organization and properties of a typical or average brain (Hagmann et al., 2008; van den Heuvel & Sporns, 2011). It is often the case that group-representative networks are generated by aggregating network data from many subjects while preserving those properties that are consistently expressed at the subject level (de Reus & van den Heuvel, 2013; Roberts, Perry, Roberts, Mitchell, & Breakspear, 2017; Zalesky et al., 2016). This approach, when performed carefully, can theoretically enhance signal while suppressing noise and artifacts, affording a clearer view of the brain’s network organization.

**Figure 1.**
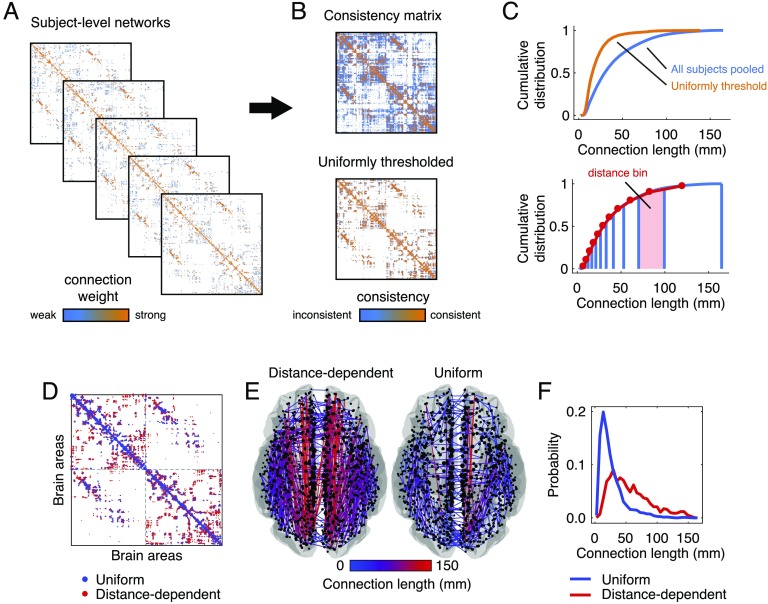
Construction and superficial comparison of group-representative matrices. Group-representative connectivity matrices summarize subject-level network data (A) by retaining features that are consistently expressed across subjects. In most applications the features of interest are the edges between brain areas and their weights. The most straightforward approach for generating a group-representative matrix involves first constructing a *consensus matrix* (B), whose elements denote the fraction of all subjects in which edges are expressed. Group-representative matrices can be estimated by retaining all connections expressed in at least τ subjects and populating those connections with weights. Though this approach is common, it suffers from a number of shortcomings. In general, because the probability of observing any given short-range connection is greater than the probability of observing a long-range connections, short-range connections also appear more consistently across subjects. As a result, imposing a *uniform consensus-based threshold* across all elements of the consensus matrix will result in a group-representative matrix in which short-range connections are expressed with much greater frequency than any single subject (C). To circumvent this issue, we present a simple alternative approach. Briefly, this involves dividing all connections into *m* bins according to their length and, within each bin, retaining the connection that is most frequently expressed. This *distance-dependent consensus-based thresholding* approach results in networks with almost the exact same edge length distribution as the typical subject. We also show differences in the group-representative matrices generated using the distance-dependent and uniform consensus-based threshold (here, we choose τ for the uniform method such the resulting matrix has a number of connections equal to that of the average subject). (D) Connections present only in the uniform method are depicted in blue and those present only in the distance-depdendent method are shown in red. (E) We plot these same method-specific connections on the brain, and color them according to their length (in millimeters). (F) In general, the connections unique to the uniform method are short range (blue curve) while those unique to the distance-dependent method include long-distance connections.

Most methods for constructing group-representative networks are variants of “consensus-based thresholding.” That is, a sparse group network is generated by specifying a threshold whose value ranges between 0 and 1, and retaining connections that are observed in at least some fraction of subjects, τ. The retained connections are usually associated with a weight while all others are set to 0 (Roberts et al., 2017) (Figures 1A, 1B). In almost every application, the same consensus threshold is applied uniformly over all possible connections. This so-called uniform consensus-based thresholding is common and group-representative networks generated using this approach appear frequently in the network neuroscience literature. It is critical to note, however, that the “correct” threshold is generally unknown and is often selected according to heuristics. Moreover, the very act of thresholding can introduce biases and cloud subsequent interpretations of network organization and statistics (Garrison, Scheinost, Finn, Shen, & Constable, 2015).

Group-representative networks are intended to serve as exemplars by preserving features consistently expressed at the level of individual subjects while reducing the level of noise and uncertainty. Among the most salient features of subject-level structural brain networks is the dependence of their topological features on their spatial embedding (Stiso & Bassett, 2018). Both the probability that two brain areas are connected and the weight of that connection, should it exist, decay monotonically with interareal distance. This effect has been reported in human structural networks reconstructed from diffusion MRI with tractography algorithms (Betzel & Bassett, 2018; Roberts et al., 2016; Samu, Seth, & Nowotny, 2014), as well as networks reconstructed using invasive methods, such as tract-tracing (Ercsey-Ravasz et al., 2013; Horvát et al., 2016), suggesting that these dependencies are not simply artifacts of any specific network construction approach, but an evolutionarily conserved feature of large-scale brain networks (van den Heuvel, Bullmore, & Sporns, 2016).

The preference for strong, short-range connections can be explained parsimoniously by cost-reduction mechanisms (Bullmore & Sporns, 2012; Vértes et al., 2012). Intuitively, longer connections are more costly; they require additional material to form and extra energy for sustained use compared with short-range connections. As a consequence, nervous systems have evolved to favor shorter, low-cost connections. Despite this, brain networks still exhibit some long-distance connections (Betzel, Medaglia, & Bassett, 2018). It is generally understood that longer connections play critical functional roles in order to offset their cost, though their precise function is still not fully understood.

Whatever their precise functional role, long-distance connections are arguably one of the most important subject-level features to preserve in any group-representative network. They play an important role in increasing shortest-path efficiency (Kaiser & Hilgetag, 2006) and engender diverse network dynamics and information processing (Betzel & Bassett, 2018). However, uniform consensus-based thresholding can produce networks that vastly underestimate the number of observed long-distance connections. This bias emerges because the consensus of connections across subjects is, itself, distance-dependent, with short-range connections appearing more consistent than longer-range connections. As a consequence, for a given consensus threshold the distribution of suprathreshold connections will always favor short-range connections at the expense of long-distance connections. That is, group-representative networks generated using a uniform consensus-based thresholding procedure will exhibit more short-range connections and fewer long-distance connections than the typical subject (Figures 1C–F). Because long-distance connections are responsible for driving certain network statistics, these group-representative networks will also fail to match subject-level networks in terms of those metrics.

Here, we present an alternative method for constructing group-representative networks. Our approach, which we haved used in previous papers (Betzel & Bassett, 2018; Betzel, Gu, Medaglia, Pasqualetti, & Bassett, 2016; Betzel et al., 2017; Mišić et al., 2015) but never appropriately benchmarked, builds upon the consensus-based thresholding framework; rather than imposing a threshold uniformly over all connections, we allow our threshold to vary as a function of distance, retaining the most consistent connections conditional upon their length. In contrast to existing approaches, we derive the distance-dependent threshold nonparametrically so as to match the pooled edge length distribution of subject-level data. We compare networks generated using this distance-dependent thresholding procedure with those generated using more traditional methods and show that, across a wide range of network statistics and comparative measures, networks generated using the distance-dependent approach outperform others. The distance-dependent procedure successfully recapitulates many of the important organizational features of subject-level networks and demonstrates promise for future exploratory studies of structural brain networks.

## RESULTS

In this section we compare four different approaches for generating group-representative structural connectivity networks.

•  Connections are retained if they appear in at least one subject. We refer to this as the “Simple” method.
•  Connections are retained if they appear in at least 50% of subjects. We refer to this as the “τ = 0.5” method.
•  Connections are retained if they appear in at least τ_Avg_ subjects, where τ_Avg_ is the consensus threshold that results in a binary density equal to that of the typical subject. We refer to this as the “τ = Avg” method. Note: We calculate this threshold separately for inter-/intra-hemispheric connections.
•  Connections are retained using a distance-dependent consensus threshold. The resulting network preserves, approximately, the edge length distribution of the typical subject. We refer to this as the “Dist.” method. As with the “τ = Avg” method, the distance-dependent threshold is introduced separately for inter-/intra-hemispheric connections.

This section is further divided into four subsections. In the first two subsections, we compare statistics of group-representative networks with those of individual subjects. In the next subsection, we characterize connectivity patterns of the group-representative matrices with respect to cognitive systems and discuss implications for our understanding of brain function. In the final subsection, we characterize how hubs are redistributed depending upon the approach used for generating group-representative brain networks.

Throughout this section, we report results of analyses using the high-resolution parcellation of the Lausanne dataset (*N* = 1,000 nodes), where white matter fiber tracts are reconstructed from diffusion spectrum imaging data using deterministic streamline tractography (See the Materials and Methods section for processing details). These results are representative of our findings using coarser parcellations. Those additional results are included in the Supporting Information, Figure S1 and Figure S2 (Betzel, Griffa, Hagmann, & Mišić, 2019). Finally, we also compare the results of the distance-dependent consensus method with a weight-based thresholding approach in which we generate a new group-representative network by imposing a weight threshold on the “Simple” group-representative network. These results of this analysis are included show in the supplement (Figure S3; Betzel, Griffa, et al., 2019).

### Uniform and Distance-Dependent Consensus-Based Thresholding Generate Systematically Different Networks

The presence and weights of edges in structural connectivity networks exhibit spatial dependencies because of cost-reduction principles and reconstruction artifacts that cause short-range connections to be more consistently expressed across subjects. As a consequence, procedures for generating group-representative networks that retain connections using uniform consensus thresholds will necessarily overestimate the number of short-range connections in a network.

In the following subsections we explore the implications of these biases in greater detail. Here, we simply show that uniform consensus thresholds generate group-representative networks with different spatial statistics than those generated using distance-dependent consensus thresholds, wherein the threshold for edge retention varies as a function of Euclidean distance. In Figure 1D, we show an adjacency matrix containing connections that are present in either the uniform or distance-dependent group-representative matrix but not both. Alongside this panel and in Figure 1E, we plot these same connections in anatomical space and color connections according to their lengths, with long/short connections appearing bright red/dark blue. In the left and right subpanel we show connections that are present in distance-dependent method but not the uniform method and vice versa. Note that the connections shown in the left subpanel tend to be long (red), indicating that the distance-dependent method retains long-distance connections that are not preserved in the uniform method. Conversely, the connections in the right subpanel tend to be short (blue), indicating that the uniform method retains short-range connections not observed in distance-dependent method. These trends can be summarized by examining the distribution of connections present in one method but not the other. In Figure 1F we show that, as expected, connections retained exclusively by the uniform method are sharply peaked around 25 mm, whereas the connections retained by the distance-dependent method are more broadly distributed and include many long-distance connections. We note that the τ = 0.5 and distance-dependent methods have equal binary density, making a direct comparison of those matrices appropriate.

These observations about differences in the connection length distributions of group-representative brain networks, though superficial, have important practical consequences for the structural properties of those networks. An overexpression of short-range connections could result in excessively cohesive brain network modules (Roberts et al., 2016; Samu et al., 2014), missing out on potentially rich intermodular connectivity patterns (Betzel, Medaglia, et al., 2018). Conversely, group-representative networks that overexpress long-distance connections may lack segregative network properties such as local clustering (Sporns & Zwi, 2004). These observations are in line with the fact that, in general, network properties are not independent of one another, and variation in one property has implications for others (Rubinov, 2016). Here, and throughout this paper, we argue that mischaracterizations of edge length distributions have profound implications for the spectrum of network properties that are exhibited by group-representative networks and whether those properties are in line with those of individual subjects’ networks.

### Consistency-Based Thresholding Does Not Preserve Subject-Level Network Statistics

There are many criteria by which group-representative connectivity matrices could be evaluated and judged. Arguably among the most important is their ability to recover and recapitulate the topological properties of the subject-level data that they supposedly represent. In this section we compare four approaches for generating group-representative networks according to how well each matches individual subjects in terms of an ensemble of network statistics. We focus specifically on local statistics such as degree, strength, clustering coefficient, betweenness centrality, and edge length distribution and global statistics such as number of binary connections, total weight, mean clustering, topological efficiency, mean path length, modularity, diameter, and degree assortativity.

**Figure 2.**
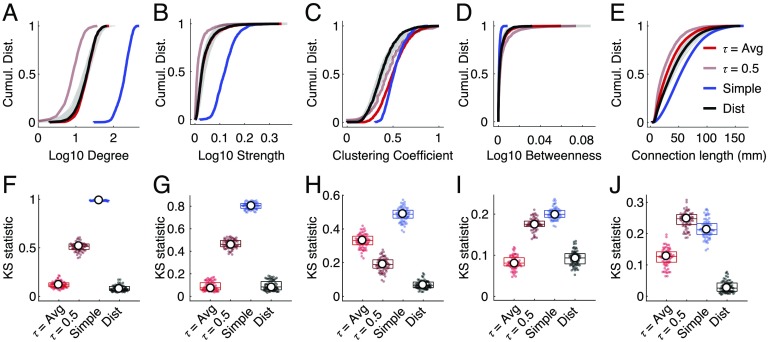
Comparing distributions across methods. We show cumulative distributions for (A) degree, (B) strength, (C) clustering coefficient, (D) betweenness centrality, and (E) connection length. Subject-level data are shown in gray. Superimposed on those distributions are curves associated with the four methods that we tested (in color). In panels (F)–(J) we show Kolmogorov-Smirnov (KS) statistics for each network measure, which compare cumulative distribution curves of methods with individual subjects.

We divided network meausures into two categories based on whether they were defined locally or globally. We compared subject-level and group-representative local measures, that is, those defined at the level of individual nodes, using Kolmogorov-Smirnov (KS) tests. The KS test statistic measures the maximum distance between two cumulative distributions, and therefore smaller values indicate closer correspondence. In Figures 2A–E, we overlay cumulative distributions of degree, strength, clustering coefficient, betweenness centrality, and edge length for each of the four group-representative networks on top of the cumulative distributions for individual subjects. The KS statistics comparing these distributions are plotted in Figures 2F–J.

For all five measures, we found that the distance-dependent consensus-based thresholding approach outperformed the other three methods, that is, smaller KS statistics (*p* < 0.05; Bonferroni corrected). These findings indicate that the distance-dependent method better preserved multiple node- and edge-level measures than the other methods, suggesting that network statistics computed on the other group-representative networks may be misleading, in that they are not necessarily representative of the typical subject.

**Figure 3.**
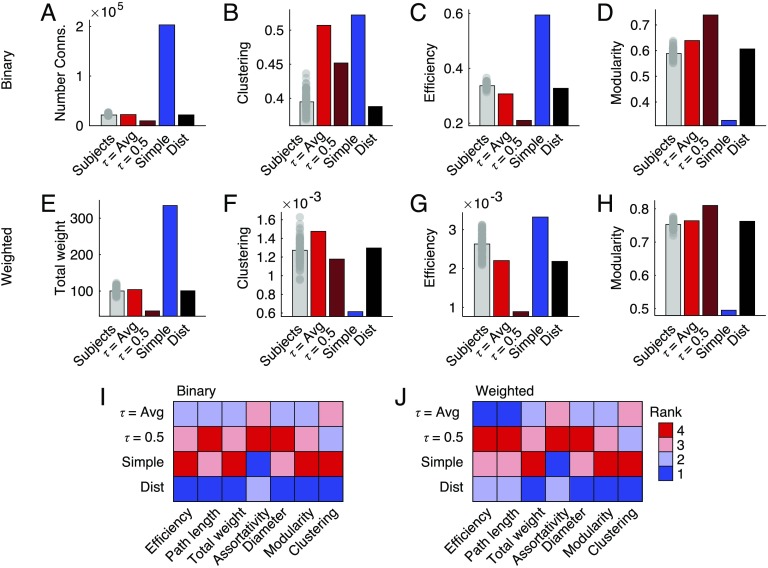
Comparing scalar network statistics. Here, we compare the performances of four different methods of group-representative brain networks to those of individual subjects. (A) Each bar represents the total number of binary connections for single subjects (gray), a uniform method with approximately the same number of connections as the average subject (bright red), a uniform method with a consensus threshold of τ = 0.5 (dark red), a “Simple” method that retains a connection if it is observed in even one subject (blue), and the distance-dependent method (black). Panels (B)–(D) show similar plots but for mean clustering, efficiency, and modularity. Panels (E)–(H) depict those same measures, but computed over weighted analogs of the binary networks. (I) For each measure shown (along with several others), we identified the method that was closest to that of the average across all subjects. In general, we find that the distance-dependent method consistently outperforms or performs comparably to the other tested methods, achieving rank 1 or 2 across all metrics.

We performed similar comparisons of the global network measures. Here, rather than comparing distributions using the KS test, we z-scored the measures computed on the group-representative networks against the corresponding subject-level distributions. A z-score close to 0 implied that the group-representative network was close to the mean subject-level value for a given network measure. In Figures 3A–H, we show binary and weighted analogs of the total number of connections in the network, mean clustering, efficiency, and modularity. We found similar results when comparing network diameter, assortativity, and mean path length (Figures 3I, 3J).

As with the local network measures, these findings suggest that decisions about how to generate a group-representative connectivity matrix have implications for its topological organization. Importantly, the most popular approach—uniform consensus-based thresholding—preserves a greater number of short-range connections compared with the typical subject and, as a result, exhibits topological properties that are inconsistent with those exhibited by that subject.

### Implications for Structure-Function Relationships

**Figure 4.**
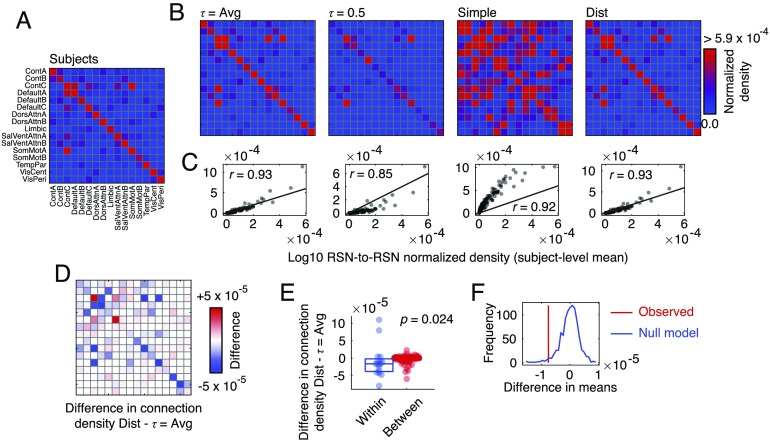
Comparing within-/between-RSN connectivity patterns. We compared different group-representative networks in terms of connection densities within and between canonical brain systems taken from Schaefer et al. (2017). (A) Inter-RSN connection density of the typical subject. (B) Inter-RSN connection densities for four different group-representative networks: (from *left* to *right*) uniform consensus method with same density as subjects, uniform consensus method with threshold set at τ = 0.5, “Simple” method, and distance-dependent method. (C) We show the correlation patterns of inter-RSN densities for each method (*y*-axis) with that of the subject average (*x*-axis). Of the methods compared here, the distance-dependent and the uniform method with same density as the typical subject performed the best. We compare these methods so as to better understand their differences. (D) Difference in inter-RSN connection density between distance-dependent and τ = 0.5 threshold methods. Blue colors indicate that connection density is greater in uniform method while red density indicates that connection density is greater in distance-dependent method. (E) We find that, on average, the uniform method results in weaker within-RSN density than the distance-dependent method, while the distance-dependent method has greater between-RSN density. (F) We show the observed difference in within- and between-RSN density and compare it against a null method. Here, we show the null distribution (blue) and the observed value (red). The null distribution was constructed by independently and randomly permuting rows/columns of each original connectivity matrix and reaggregating according to the RSN system labels. Then we compute the mean difference of within-/between-RSN densities.

In the previous section, we compared group-representative networks in terms of how well they recapitulated topological properties of subject-level networks. Another important dimension along which group-representative networks can be compared is in terms of how their structural connections map onto the brain’s functional and cognitive systems. Here, we explore this structure-function relationship by averaging structural connectivity weights among previously described cognitive systems—resting-state networks (RSNs; Schaefer et al., 2017). Resting-state networks are composed of brain areas with similar *functional* connectivity profiles and recapitulate the collections of brain areas that coactivate during cognitively demanding tasks. We compute inter- and intra-RSN connectivity density for each of the four group-representative methods, in each case generating a system-by-system connection density matrix (Figure 4B). We also do the same for individual subjects, averaging over these subject-level matrices to obtain a single matrix whose elements represent the mean intersystem connection density across all subjects (Figure 4A). To compare group and subject matrices, we compute the Pearson correlation of their upper triangle + diagonal elements.

In general, we find that each group-representative matrix is positively correlated with the subject-level matrix, indicating that, overall, system-to-system connectivity at the subject level is preserved at the group level by all methods. Nonetheless, there is considerable variability across group-representative methods in terms of correlation magnitude and deviation from the identity line (Figure 4C). For instance, the “Simple” method exhibited a correlation of *r* ≈ 0.92 but massively overestimated the amplitude of connection densities. Similarly, the uniform method with a threshold of τ = 0.5 exhibited a much weaker correlation of *r* = 0.85. The remaining two methods, on the other hand, exhibited much stronger correlations with magnitudes in excess of *r* ≈ 0.93 and approximately the same spread of data points around the identity line.

The two best-performing methods were the uniform method for which we selected a threshold resulting in the same density as the average subject and the distance-dependent method. We compared these two methods in greater detail to better understand the implications of using one method versus the other. First, we computed the difference in inter-RSN connectivity density (Figure 4D). We found that there were subtle yet systematic differences. In particular, we found that the distance-dependent method exhibited much weaker within-RSN density compared with that of the uniform method while also exhibiting stronger between-RSN connection density (*p* < 0.05, permutation test; Figures 4E, 4F).

These findings have important implications for the analysis and interpretation of brain network data. This is especially true for studies that aim to link features of structural and functional brain networks to one another. Past studies using group-representative data constructed using a uniform consensus threshold may fail to match the specificity of subject-level networks, while the simple averaging procedure may overestimate the weights of short-range connections (van den Heuvel & Sporns, 2013). These failings could, in principle, lead to mischaracterizations of structure-function associations.

More importantly, these findings suggest that differences in the construction method for group-representative networks can result in networks that emphasize either segregative features—that is, stronger within-RSN connection densities, as expressed by the uniform method—or integrative features—that is, stronger between-RSN connection densities as expressed by the distance-dependent method. The balance between information segregation and integration is thought to be an important organizational principle responsible for shaping brain network topology (Cohen & D’Esposito, 2016; Deco, Tononi, Boly, & Kringelbach, 2015; Sporns, 2013). Our findings indicate that different group-representative methods differentially emphasize these characteristics, indicating that a user’s seemingly arbitrary choice in method can have implications for measures made on a network.

### Hub (Re)distribution

A third means of comparing group-representative networks against one another is to measure the redistribution of hub areas, that is, assessing changes in the locations of “central” brain areas as a result of choosing one group method versus another. Here, we compare the spatial distribution of betweenness centrality, node degree, clustering coefficient, and participation coefficient under uniform and distance-dependent methods. To ensure that comparisons are as fair as possible, we rank-transformed all measures prior to comparison.

**Figure 5.**
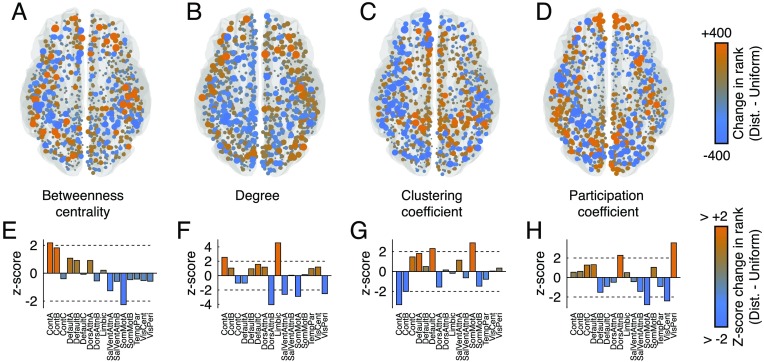
Comparing spatial distribution of hubs. We compare four measures of hubness: (A) betweenness centrality, (B) degree, (C) clustering coefficient, and (D) participation coefficient. Rather than compare raw values, which can fluctuate because of small differences in global network properties like total number of connections or weight, we compare ranked values of each measure and observe whether a node’s rank is smaller/greater under the distance-dependent or uniform method. Orange-colored nodes indicate that a node’s value is greater under the distance-dependent method than it is under the uniform method. Blue-colored nodes indicate the opposite. We then aggregated node-level differences in ranked measures by cognitive systems and compared the mean system-level values with those obtained under a null method. In panels (E)–(H) we show the z-scored system means. In general, large-magnitude z-scores indicate bigger greater system-level differences between the two methods.

In general, we found widespread and hemispherically symmetric redistribution of hub regions. In the case of betweenness centrality (Figure 5A), we found that under the distance-dependent method, areas associated with cognitive control are increasingly central, while areas in the somatomotor system become less central (*p* < 0.05; corrected for multiple comparisons by controlling false discovery rate at 5%; Figure 5E). In terms of degree (Figure 5B), we find that control and limbic systems make a greater number of connections, while dorsal attention, salience/ventral attention, somatomotor, and visual systems exhibit fewer connections (Figure 5F). For clustering coefficient (Figure 5C), we find that components of default mode and motor systems are more clustered while multiple components of the control network are less clustered (Figure 5G). Finally, in terms of participation coefficient (Figure 5D), we find that the dorsal attention and visual systems exhibit greater participation, whereas somatomotor and other visual systems exhibit decreased participation (Figure 5H).

These findings have important implications for our understanding of brain function. Hubs and central brain areas are believed to be important for controlling interareal communciation and regulating the flow of information within and between brain network modules. Indeed, the designation of an area as a “hub” has been important for hypothesis generation and has also played an important confirmatory role in other studies. Our findings suggest that these definitions are, at least to some extent, dependent upon the method used to generate a group-representative network. Moreover, some of the most salient differences between methods appear localized to specific cognitive systems, which has additional implications for how we interpret findings related to hubs and brain function.

## DISCUSSION

In this paper we flesh out the details of a new method for generating group-representative brain networks that outperforms more conventional methods in terms of preserving subject-level statistics. Specifically, we show that this method better preserves local and global network statistics, that its structure-function relationships are more consistent with those of individual subjects, and that it gives rise to a different intuition of where highly central hub regions are located in the brain.

### Structural Networks Need Long-Distance Connections

Here, we found that compared with uniform consensus-based thresholding, a distance-dependent threshold preserves to a greater degree the connection length distributions observed in individual subjects. We argue that this difference in connection length distributions has both practical (i.e., measurable) and theoretical consequences. Practically, we show that expressing fewer long-distance connections results in networks that are more clustered and, as a result of increased rate of triadic closure, more modular than that of the typical subject (Henderson & Robinson, 2013). Similarly, lacking long-distance shortcuts results in networks that are less efficient and that possess longer characteristic path length than the average subject (Henderson & Robinson, 2013; Samu et al., 2014). Overall, the uniform method results in networks that emphasize segregative traits at the expense of those that support integration of information (Sporns, 2013). This is confirmed further when we compared the intersystem connection densities of the distance and uniform methods, observing that within-community density was less, on average, using the distance method compared with the uniform method. Additionally, the differences in the features preserved by each method contribute to shaping the spatial distribution of hubs across the brain. Overall, these findings suggest that the principal advantage of the distance-dependent method is that it better preserves network features that emphasize information integration.

The presence of long-distance connections, though useful in theory for brain network function, also presents a methodological challenge and their inclusion in network models demands careful attention. For instance, recent studies have shown that in highly modular networks, the addition of spurious long-distance intermodular connections induce larger changes in network statistics like clustering, modularity, and efficiency compared with the addition of short-range, intramodular connections (Zalesky et al., 2016). The results of our study, on the other hand, suggest that by including long-distance connections, we better recapitulate the network properties of individual subjects. This discrepancy between these findings is a direct result of two distinct sets of assumptions: in Zalesky et al. (2016), the authors treat the “true” network to be one generated using uniform consensus-based thresholding with τ = 0.5, resulting in a highly modular network, whereas we treat the “true” network to be one with statistics similar to that of the typical subject, which is less modular, less clustered, and more efficient. In general, the organization of the ground-truth connectome remains unknown, and it is unclear which set of assumptions is more appropriate. With advances in cellular-level reconstruction, it may soon be possible to obtain a clearer picture of true structural connections, which could be used to inform out macroscale models of connectivity and resolve this debate (Briggman, Helmstaedter, & Denk, 2011; Helmstaedter et al., 2013).

### The Role of Group-Representative Network Analysis

In this study we focus on group-representative networks. Analysis of these group networks has been and remains an important component of network neuroscience. In the case of nonhuman datasets, group network analysis is almost always performed out of necessity. Invasive methods like tract-tracing limit the number of experiments that can be performed on any one animal brain. As a result, whole-brain networks are necessarily composites of many animals (Markov et al., 2012; Noori et al., 2017; Oh et al., 2014). Human structural networks constructed from diffusion MRI data using tractography algorithms are sensitive to scan parameters and prone to false positives and negatives (Maier-Hein et al., 2016; Reveley et al., 2015). Analyses of human networks, therefore, benefit from aggregation of multisubject cohorts into a group-representative network, which serves to enhance signal while reducing the level of noise and uncertainty. The resulting networks can be treated as exemplars and used to uncover key structural traits and organizing principles (Hagmann et al., 2008; van den Heuvel & Sporns, 2011), as the basis for dynamic methods (Mišić et al., 2015), and serve as a sort of “prior” for other machine and statistical learning approaches (Rosenthal et al., 2018).

However, analysis and interpretation of group-level networks presume that those networks are, in fact, representative of the typical subject. Group networks that violate this assumption can contribute misleading or inaccurate insight into brain network organization and function. We show here that group-representative networks constructed using a uniform consensus-based threshold, which fail to preserve important spatial properties of subject-level brain networks, may be especially susceptible to such inaccuracies. Our work suggests that the uniform method generates networks that tend to overestimate the cohesiveness of communities. In addition, the uniform method also presents a conflicting account of hub distributions throughout the brain when compared with the distance-dependent method. Because analysis of group-representative networks remains a powerful approach, understanding and accounting for their limitations and biases should be investigated in future research.

### Limitations

Here, we present a method for constructing group-representative networks, demonstrating that this approach results in group networks that better preserve subject-level properties than existing approaches. Nonetheless, our study suffers from some limitations.

First, we make the overarching assumption that the long-distance connections observed in single-subject networks (which we preserve in our group-representative network) are “real” and not strictly artifacts of the tractography algorithm. This assumption is supported, first, by the fact that long-distance connections are, in general, more challenging to reconstruct using common tractography parameters. Completing long streamlines requires strong spatial coherence of the diffusion field over distances greater than 150 mm, which is unlikely to occur in the presence of high background noise (Jones, Knösche, & Turner, 2013). Second, long-distance connections, typically, do not appear randomly distributed, but are clustered (Betzel & Bassett, 2018). That is, if regions *i* and *j* are connected by a long-distance tract, it is likely that other regions in *i*’s spatial neighborhood are connected to *j* and *j*’s spatially proximal neighbors (and vice versa). These observations suggest that long-distance connections cannot easily be explained as errant “one-off” reconstructions. Nonetheless, tractography has known shortcomings (Maier-Hein et al., 2016; Reveley et al., 2015), and the verisimilitude surrounding long connections remains unclear. Advances in hardware, fiber reconstruction software (Pestilli, Yeatman, Rokem, Kay, & Wandell, 2014), and detailed comparisons of tractography with tract-tracing data (Calabrese, Badea, Cofer, Qi, & Johnson, 2015) will help future studies overcome these issues.

A second limitation concerns the network measures and metrics used to compare group-representative networks to one another and to individual subjects. These measures were selected because they emphasized network topology as well as its relationship to neuroscientifically relevant metadata (i.e., cognitive functional systems). However, these measures are, first, not necessarily an exhaustive list and it is unclear whether the distance-dependent method’s performance would be better than other methods were we to select a different set of measures. Second, network measures tend to be correlated with one another—for example, a network with high efficiency will tend to have short path length. Therefore, the comparisons we made were not necessarily independent of one another. Though we intentionally selected a wide range of measures to help address these issues, our analyses could be extended in future work to include an even broader range of measures and comparative metrics.

A third limitation concerns the applicability of the distance-dependent thresholding procedure (or any consensus-based thresholding procedure for that matter) to fully weighted connectivity matrices, like those generated from probabilistic tractography or functional connectivity data. One strategy to extend our approach is to first impose a threshold on subject-level fully weighted matrices, which is fairly common in the analysis of the aforementioned data types (Fallani, Latora, & Chavez, 2017; Gollo et al., 2018). With sparse subject-level data, the consensus-based thresholding procedures can be carried out as reported here. Generating consensus matrices that, themselves, are fully weighted remains an outstanding challenge. In that case, consensus-based thresholding fails (all connections are present across all subjects). Moreover, in the case of functional connectivity, which is usually estimated as a correlation matrix, special care must be taken to preserve statistically defined transitive relationships (Zalesky, Fornito, & Bullmore, 2012). Future work should be directed to more explicitly investigate consensus methods for full matrices.

Yet another limitation concerns the ability of modern tractography methods for reconstructing long-distance tracts. We show that traditional consensus-based thresholding methods prune away long-distance streamlines in single subjects because the locations of those tracts are less consistent across subjects than short-range tracts. Here, we interpret the reduction in long-distance tracts (which are expressed in every subject) as evidence of a failing in consensus-based thresholding procedures. Another interpretation, however, is that long-distance tracts appear less consistent because they are spurious. We argue that this is likely not the case, as long-distance connections (though inconsistent) nonetheless exhibit high levels of clustering and structure (Betzel & Bassett, 2018), suggesting that they are distributed in a far from random manner. This issue should be investigated in more detail in future work.

In this study, we generated group-representative networks by retaining connections with the greatest consensus across subjects, that is, those that were expressed in a large fraction of individuals. This ensures that group matrices preserve those connections most consistently expressed across subjects rather than the connections with the strongest weights (another common procedure for denoising network data; Fallani et al., 2017; van Wijk, Stam, & Daffertshofer, 2010). Moreover, it is expected that connection weights vary over several orders of magnitude (Buzsáki & Mizuseki, 2014). This provides some rationale for retaining connections with high consensus across subject irrespective of their weight. In contrast, imposing a weight-based threshold across connections effectively truncates a heavy-tailed distribution and restricts connection weights to a narrower regime. Nonetheless, there may be scenarios in which weight-based thresholding is more appropriate for constructing group-representative networks, for example, when the noise level is exceptionally high and there is poor consensus across subjects. Much additional work is needed to identify the situations where one or the other approach should be preferred.

A final limitation is the necessity that the subject-level matrices used to estimate the group-representative network be sparse. Both the uniform and the distance-dependent methods rely on the intuition that some connections are more common across individuals than others. For some diffusion MRI and tractography algorithms—such as probabilistic tractography—this is not always the case (Descoteaux, Deriche, Knosche, & Anwander, 2009). Nonetheless, it may be possible to adapt the approaches used here with sparse deterministic tractography to the probabilistic case by substituting connection probability measures for the consensus. Care would have to be taken to deal with the potentially confounding geometric and spatial biases (Roberts et al., 2016). Future work should investigate this in greater detail.

### Conclusion

Overall, our findings suggest that care must be taken when studying and analyzing group-representative networks. We presented an approach for limiting discrepancies between subject- and group-level networks by adding a distance-dependence to the consensus threshold. This approach will aid in future studies that seek to investigate general properties of structural brain networks.

## MATERIALS AND METHODS

### Connectome Dataset

In this study we compared methods for constructing group-representative brain networks from structural connectivity data. We carried out these comparisons using diffusion spectrum MRI data parcellated into networks at three different organizational scales. Here, we describe those processing steps in greater detail.

#### MRI acquistion.

A total of 70 healthy participants (age 28.8 ± 9.1 years old, 43 males) were scanned on a 3T scanner with a 32-channel head coil (Magnetom TrioTim, Magnetom Prisma, Siemens Medical, Germany). The session included (a) a magnetization-prepared rapid acquisition gradient echo (MPRAGE) sequence (1 × 1 × 1.2 mm resolution, 240 × 257 × 160 voxels; TR = 2,300 ms, TE = 2.98 ms, TI = 900 ms); (b) a diffusion spectrum imaging (DSI) sequence (2.2 × 2.2 × 3 mm resolution; 96 × 96 × 34 voxels; TR = 6,100 ms, TE = 144 ms; q4half acquisition with maximum b-value 8,000 s/mm^2^, one b0 volume). Informed written consent was in accordance with institutional guidelines and the protocol was approved by the Ethics Committee of Clinical Research of the Faculty of Biology and Medicine, University of Lausanne, Switzerland.

#### MRI preprocessing.

The individual connection matrices were computed using the open aggregation software Connectome Mapper (http://www.connectomics.org; Daducci et al., 2012), which calls different tools at different processing steps using the parameters described in the sequelae.

MPRAGE volumes were segmented into white matter, gray matter, and cerebrospinal fluid using FreeSurfer software version 5.0.0 (Dale, Fischl, & Sereno, 1999). Cortical volumes were segmented into five progressively finer parcellations, with 68, 114, 219, 448, and 1,000 approximately equally sized parcels (Cammoun et al., 2012). Here, we analyze the 68-, 219-, and 1,000-parcel divisions. DSI data were reconstructed following the protocol described by Wedeen and colleagues (V. J. Wedeen, Hagmann, Tseng, Reese, & Weisskoff, 2005), thus estimating an orientation distribution function (ODF) in each voxel. Up to three main streamline orientations were idenntified in each voxel as the maxima of the ODF (Diffusion Toolkit software, http://www.trackvis.org/dtk).

Structural connectivity matrices were estimated for individual participants using deterministic streamline tractography on reconstructed DSI data, initiating 32 streamline propagations per diffusion direction per white matter voxel (V. Wedeen et al., 2008). The MPRAGE and the brain parcellation were linearly registered to the subject diffusion space (b0) using a boundary-based cost function (FreeSurfer software; Greve & Fischl, 2009). For each starting point, streamlines were grown in two opposite directions with a fixed step size equal to 1 mm. As the streamline entered new voxels, growth contributed along the ODF maximum direction that produced the least curvature. Streamlines were terminated if changes in direction were greater than 60 deg/mm. Tractography completed when both ends of the streamline left the white matter mask. Structural connectivity between pairs of parcels was estimated in terms of streamline density, defined as the number of streamlines between two parcels normalized by the mean length of the streamlines and the mean surface area of the parcels.

### Single-Subject Networks and Connection Consensus

Let **A**_*s*_ ∈ ℝ^*N*×*N*^ be the weighted and symmetric structural connectivity matrix for subject *s* = 1, …, *T*, whose element *A*_*ijs*_ indicates the normalized streamline count between brain areas *i* and *j*. Given the set of matrices 𝒜 = {**A**_*s*_} we can calculate the consensus matrix, **C** ∈ ℝ^*N*×*N*^, whose element *C*_*ij*_ = $\frac{1}{T}\sum_{s=1}^{T}$[*A*_*ijs*_ > 0]. Intuitively, then, *C*_*ij*_ ∈ [0, 1] indicates the fraction of *T* subjects for whom the connection {*i*, *j*} is expressed.

### Group-Representative Network Construction

In this paper we compare several strategies for constructing group-representative networks for structural connectivity estimated from dMRI and reconstructed using tractography. In this section, we introduce several approaches for doing so.

#### Simple average.

The most naïve approach for generating a group-representative connectivity matrix is to let each connection’s weight be its mean value over all subjects, ignoring those for whom a connection is not expressed, that is, *A*_*ijs*_ = 0. We refer to this approach as the *simple average* and denote the estimated group-representative connectivity matrix as **A**^*simp*^, whose elements are defined as $A_{ij}^{simp}=\frac{1}{\left|T_{>0}\right|}\sum_{s\in T_{>0}}A_{ijs}$. Here, *T*_>0_ is the set of subjects satisfying *A*_*ijs*_ > 0.

#### Consistency-based thresholding.

A more common approach for generating group-representative matrices is to impose a threshold, τ, over the elements of a consensus matrix, **C**, so that the only elements retained are those that satisfy *C*_*ij*_ ≥ τ. The intuition is that a good group-representative matrix should preserve the features, in this case connections, that are consistently expressed across individual subjects. Within the broader consensus-based thresholding framework there are many strategies for implementation. In this section we hightlight those that are explored in this paper.

The most common variant of consensus-based thresholding is the imposition of a *uniform* threshold over all connections. That is, all possible elements of the consensus matrix are considered simultaneously and those that survive *C*_*ij*_ ≥ τ are retained. In contrast, a *restricted* threshold is one in which connections are grouped into *K* classes according to some criteria and a class-dependent threshold, τ(*k*), where *k* ∈ {1, …, *K*}, is imposed separately over each class. For example, connections could be classified according to whether their starting and termination points fall within the same or different hemispheres. The restricted threshold is not limited to ordinal data, but can also be used with continuous variables through discretization. Interareal distance, for instance, is a continuous variable that measures the Euclidean distance between areal centroids. One could impose a distance-dependent threshold, τ(*D*), by discretizing the interval of possible Euclidean distances into nonoverlapping bins. Each bin would include connections that span a particular range of distances, and distinct thresholds could be imposed within each bin.

Note, for all group-representative networks generated using any of the three consensus-based thresholding procedures, connection weights were determined using the following two-step process. First, each retained connection was assigned its corresponding weight from the “simple average” connectivity matrix. Once all connections had an assigned weight, we reassigned weights from the pooled subject-level connection weights *via* linear interpolation. Thus, the resulting matrices had connection weight distributions approximately identical to that of the typical subject.

#### Models tested in this submission.

Here, we tested four different methods for generating group-representative networks. The first was the “Simple” method, which retained a connection and its average weight if it was observed in at least one subject. The second and third methods were variants of the uniform consensus-based threshold method. The first of these imposed a threshold of τ = 0.5 over all connections, so that the group network included only those connections expressed in at least half of the subject cohort. The second method involved choosing a consensus threshold such that resulting network had a binary density as close as possible to that of the average subject. We refer to this method as the τ = Avg method. The fourth and final method was a distance-dependent threshold. In this method, subjects’ edge lengths are combined. Given this list of edges and their lengths, we define *M* bins based on edge length percentiles where *M* is the total number of edges in the consensus network. Next, and for each bin, we identify all possible edges who, based on their lengths, fall into that bin. Of those possible edges, we choose the one with the greatest consensus across subjects. If there exists a tie, we choose the edge with the greatest weight on average. This procedure generates a network with approximately the same edge length distribution of the original network while still selectively preserving edges with high consensus across subjects.

We note that, in general, there are many thresholding procedures with different aims and different criteria for retaining or pruning a connection. Here, we focused on some of the most common thresholding procedures, all of which operated upon the consensus matrix, **C**. Specifically, the τ = 0.5 method can be regarded as a realization of the common “absolute thresholding” procedure, whereas the τ = Avg realizes a “proportionality threshold.” While these procedures are common in the network neuroscience literature, they are generally applied to matrices whose elements represent connection weight, such as fiber density, correlation magnitude, fractional anisotropy, and so on. Here, because our goal is dually to threshold a matrix but also to retain features that are common across subjects, we apply these thresholding methods to a consensus matrix rather than a weight matrix. It remains unclear whether weight-based thresholding is an appropriate method for generating group-representative matrices. This should be investigated more explicitly in future work.

We note that of the methods tested here, τ = 0.5 and the distance-dependent method have the same density (equal to that of the average subject’s density). The other methods, τ = 0.5 and the “Simple” method, both have different densities. This has important implications, as fluctuations in network density is known to bias many network measures. Therefore, only the τ = 0.5 and the distance-dependent methods are directly comparable.

### Network Measures

We compared subject-level and group-representative networks using a set of measures that quantify different aspects of network topology (all measures were computed using functions provided as part of the Brain Connectivity Toolbox (https://sites.google.com/site/bctnet/; Rubinov & Sporns, 2010). These measures included binary and weighted total connection weight, degree, clustering coefficient (nodal and global), betweenness centrality, efficiency, path length, diameter, multiscale modularity, participation coefficient, assortativity, and connection length. In this section, we describe those measures in greater detail.

#### Degree and total weight.

Among the simplest structural measures one can calculate given a connectivity matrix, **A** = {*A*_*ij*_}, is the degree of node *i*, which summarizes the total number (or weight) of its connections:

$$k_{i}=\underset{j}{\sum }A_{ij}.$$ (1)

Given the vector of nodes’ degrees, **k** = {*k*_*i*_}, we can then calculate the total weight of the network as 2*m* = ∑_*i*_ *k*_*i*_ (the factor of two is necessary in this case because of the undirectedness of the networks considered here).

#### Clustering coefficient.

Another simple measure is clustering coefficient of each node, *i*. The clustering coefficient measures the density of connections among all of *i*’s neighbors and is calculated as

$$c_{i}=\frac{2t_{i}}{k_{i}(k_{i}-1)},$$ (2)

where *t*_*i*_ = ∑_*j*,*h*_ *A*_*ij*_*A*_*ih*_*A*_*jh*_ is the number of triangles surrounding node *i*. The node-level clustering coefficient can be averaged to summarize the mean clustering of a network, *C* = $\frac{1}{N}\sum_{i}c_{i}$.

#### Path-based measures: Characteristic path length, diameter, betweenness centrality, and efficiency.

Given the connectivity matrix **A** = {*A*_*ij*_}, we define the matrix **D** = {*D*_*ij*_} to be the shortest paths matrix, whose element *D*_*ij*_ is equal to the length of the shortest topological path between nodes *i* and *j*. For binary networks, shortest paths are calculated in terms of geodesic distance, that is, number of steps. For weighted networks, however, shortest paths are calculated based on a transformation of edge weights to length. Here, we use a reciprocol weight-to-length transformation, that is, *L*_*ij*_ = $\frac{1}{W_{ij}}$. Once the shortest paths matrix has been calcualted, we can define characteristic path length as

$$L=\frac{2}{N(N-1)}\underset{i,j>i}{\sum }D_{ij}.$$ (3)

We can also define network diameter as

$$\Delta =\arg\max D_{ij}.$$ (4)

Finally, we define efficiency as

$$E=\frac{2}{N(N-1)}\underset{i,j>i}{\sum }\frac{1}{D_{ij}}.$$ (5)

A measure related to the concept of shortest paths is betweenness centrality. Let ρ_*hj*_ be the number of shortest paths between nodes *h* and *j* and ρ_*hj*_(*i*) be the number of shortest paths between *h* and *j* that pass through node *i*. Then the betweenness centrality of node *i*, which measures the fraction of all shortest paths that pass through node *i*, is calculated as

$$b_{i}=\frac{1}{\left(N-1)(N-2\right)}\underset{h,j,h\neq i,h\neq j,i\neq j}{\sum }\frac{\rho_{hj}(i)}{\rho_{hj}}.$$ (6)

#### Modularity maximization, and the participation coefficient.

Many real-world networks exhibit modular architecture, meaning that their nodes can be meaningfully decomposed subnetworks (also called “communities” or “modules”) that are internally cohesive but segregated from one another. Though there are many approaches for detecting modules in networks, one of the most popular is modularity maximization, which partitions each node *i* into one of *K* communities σ_*i*_ ∈ {1, … *K*} by maximizing a modularity quality function, designed by the variable *Q* (Newman & Girvan, 2004). Modularity functions have the following form:

$$Q=\frac{1}{2m}\underset{c}{\sum }\underset{i,j\in c}{\sum }[A_{ij}-P_{ij}].$$ (7)

Here, *A*_*ij*_ and *P*_*ij*_ are the observed and expected weights of the connection between nodes {*i*, *j*}. The double summation ensures that, effectively, the sum counts only pairs of nodes that fall within communities, that is, σ_*i*_ = σ_*j*_. Accordingly, *Q* is optimized when the observed density of connections within communities is maximally greater than what would be expected by chance. Here, we let *P*_*ij*_ = $\frac{k_{i}k_{j}}{2m}$, which corresponds to a chance method in which each node’s degree is preserved but where its connections are otherwise made at random.

The standard formulation of modularity, *Q*, suffers from what is known as a “resolution limit” rendering it incapable of detecting communities below some characteristic scale determined by a network’s overall density (Fortunato & Barthelemy, 2007). To circumvent this issue, a parameterized version of the modularity function exists:

$$Q(\gamma )=\frac{1}{2m}\underset{c}{\sum }\underset{i,j\in c}{\sum }[A_{ij}-\gamma P_{ij}].$$ (8)

Here, γ is the structural resolution parameter, which scales the relative contribution of the expected weight. Effectively, the value of γ determines the scale of detected communities, with small and large values of γ returning correspondingly larger or smaller communities. This parameterization does not directly address the issue of the resolution limit; it simply shifts the scale below which communities are undetectable.

Optimizing *Q*(γ) is computationally intractable. However, there exist many methods and heuristics for approximating the optimal solution. One of the most common is the so-called Louvain algorithm, which has proven both fast and accurate in benchmarking tests (Blondel, Guillaume, Lambiotte, & Lefebvre, 2008). The Louvain algorithm is stochastic, however, and its estimate of the optimal partitions depends upon initial conditions. Accordingly, it is common to repeat the algorithm many times to generate a sample of near-optimal solutions.

Here, we vary γ over a range of 0.7 to 2.1 in increments of 0.1. At each discrete value, we optimize *Q*(γ) using a generalization of the Louvain algorithm 100 times (Jutla, Jeub, & Mucha, 2011).

The partitions generated by modularity maximization and related community detection algorithms can be used to further characterize different aspects of network organization and function. One such metric is the participation coefficient, which measures the extent to which a node’s connections are distributed across modules or concentrated within its own module (Guimera & Amaral, 2005). Let κ_*i*σ_ denote the total weight of connections node *i* makes to module σ. Participation coefficient is calculated as

$$p_{i}=1-\underset{\sigma }{\sum }{\left(\frac{\kappa_{i\sigma }}{k_{i}}\right)}^{2}.$$ (9)

Intuitively, the closer *p*_*i*_ is to 1, the greater the extent that *i*’s connections are distributed across many different modules. Values of *p*_*i*_ close to 0 indicate that *i*’s connections are conncentrated within a few modules.

#### Degree assortativity.

We also computed degree assortativity, a measure that quantifies the extent to which nodes of a given degree tend to connect with other nodes of similar degree (Newman, 2002). Intuitively, degree assortativity is a Pearson correlation of the degrees at different endpoints of each edge in the network and is calculated as

$$r=\frac{M^{-1}\sum_{i}j_{i}k_{i}-[M^{-1}\sum_{i}\frac{1}{2}{\left(j_{i}+k_{i}\right)}^{2}]}{M^{-1}\sum_{i}\frac{1}{2}(j_{i}^{2}+k_{i}^{2})-{\left[M^{-1}\sum_{i}\frac{1}{2}(j_{i}+k_{i})\right]}^{2}}.$$ (10)

Here, *i* = 1, …, *M* indexes the edges in the network, and *j*_*i*_ and *k*_*i*_ are the degrees of nodes connected by the *i*th edge.

#### Connection length.

Finally, we also computed connection length distributions. We define the length of a connection between nodes {*i*, *j*} as the Euclidean distance separating the centroids of those regions. In general, connections are curvilinear and do not adhere to straight-line distances. However, in studies that measured both Euclidean distance and the curvilinear fiber lengths of connections, these measures were found to be highly correlated (Betzel, Avena-Koenigsberger et al., 2016). This implies that, while Euclidean distance is not a perfect substitute for a connection’s length, it is a very good first-order approximation.

## SUPPORTING INFORMATION

Code for generating the uniform and distance-dependent group-representative networks is available at https://www.richardfbetzel.com/code/.

## AUTHOR CONTRIBUTIONS

Richard Betzel: Conceptualization; Formal analysis; Software; Writing – original draft; Writing – review & editing. Alessandra Griffa: Data curation; Resources; Writing – review & editing. Patric Hagmann: Data curation; Resources; Writing – review & editing. Bratislav Mišić: Conceptualization; Formal analysis; Supervision; Writing – original draft; Writing – review & editing.

## FUNDING INFORMATION

McGill University, Canada First Research Excellence Fund: McGill University for the Healthy Brains for Healthy Lives initiative, Award ID: 1a-DF-1. Bratislav Mišić, Natural Sciences and Engineering Research Council of Canada, Award ID: RGPIN #017-04265. Bratislav Misic, Fonds de recherche Quebec - Sante (ChercheurBoursier) and the Canadian Institutes of Health Research, Award ID: #391300. Richard Betzel, Indiana University Office of the Vice President for Research Emerging Area of Research Initiative, Learning: Brains, Machines and Children.

## TECHNICAL TERMS

Group-representative network: A single network intended to represent the connectivity data of many subjects.
Consensus thresholding: A procedure for generating group-representative networks by retaining only those connections present in a given fraction (the threshold) of subjects.
Distance-dependent consensus thresholding: Same as consensus thresholding, but where the threshold for connection retention varies with distance.
Structural connectivity: A map of physical connections among neural elements (e.g., axonal projections among neurons/populations, white matter fiber pathways among brain areas/parcels).
Connectome: A comprehensive network map of connections among neural elements.
