# Supplementary material for: Distance-dependent consensus thresholds for generating group-representative structural brain networks
Source: Netw Neurosci. 2019 Mar 1;3(2):475–96. doi: 10.1162/netn_a_00075 (PMC6444521; doi:10.1162/netn_a_00075)
Supplement: Supplementary file 1 [file netn-03-475-s001.pdf]

Betzel, R. F., Griffa, A., Hagmann, P., & Mišić, B. (2019). Supporting information for "Distance-dependent consensus thresholds for generating group-representative structural brain networks." *Network Neuroscience*, 3(2), 475–496. https://doi.org/10.1162/netn_a_00075

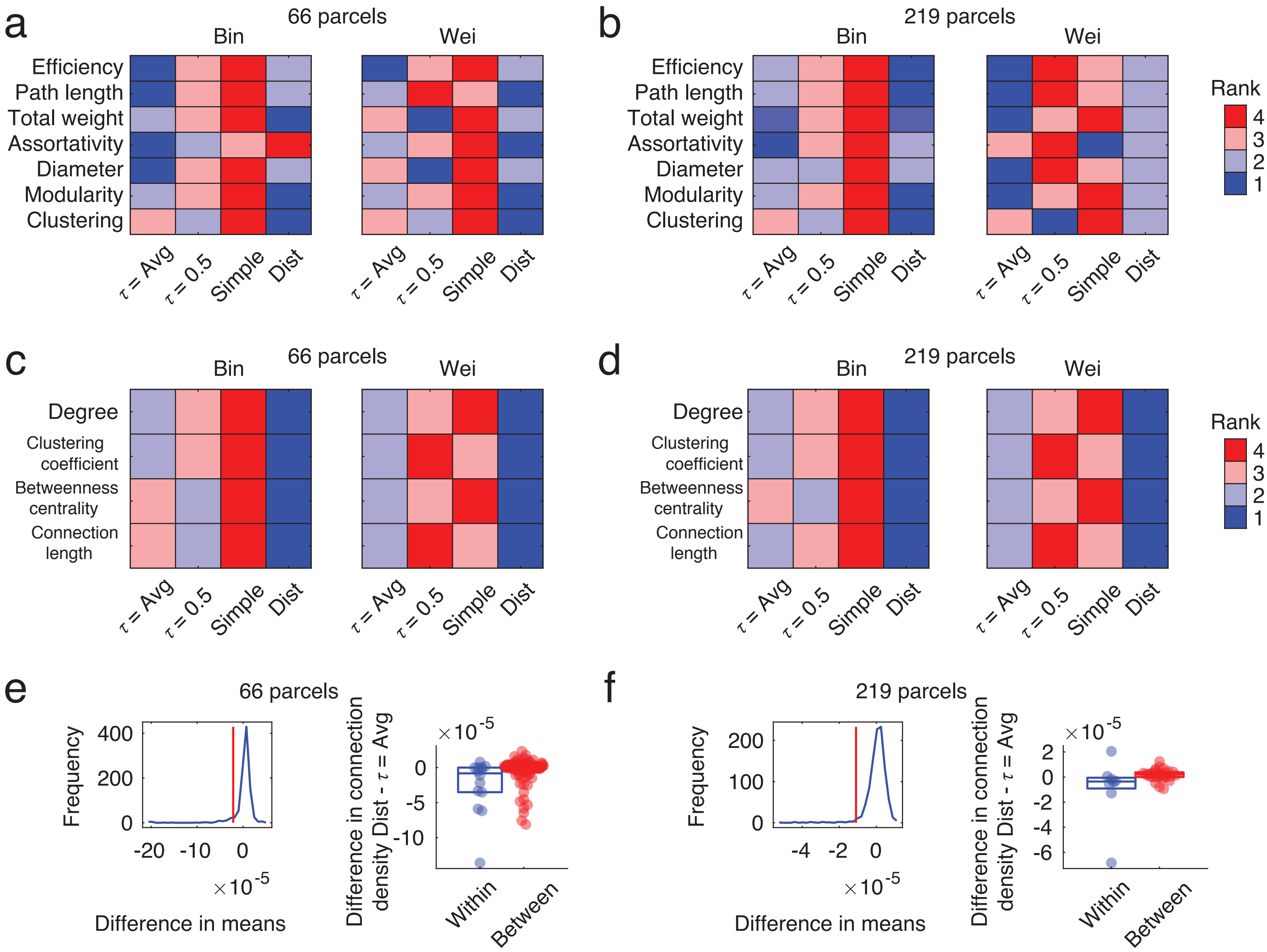

a
66 parcels
Bin
Wei
Efficiency
Path length
Total weight
Assortativity
Diameter
Modularity
Clustering
τ = Avg
τ = 0.5
Simple
Dist
b
219 parcels
Rank
4
3
2
1
c
Degree
Clustering coefficient
Betweenness centrality
Connection length
d
e
Frequency
400
200
0
-20
-10
0
×10^-5
Difference in means
Difference in connection density Dist - τ = Avg
×10^-5
0
-5
-10
Within
Between
f
200
100
-4
-2
2
-2
-4
-6

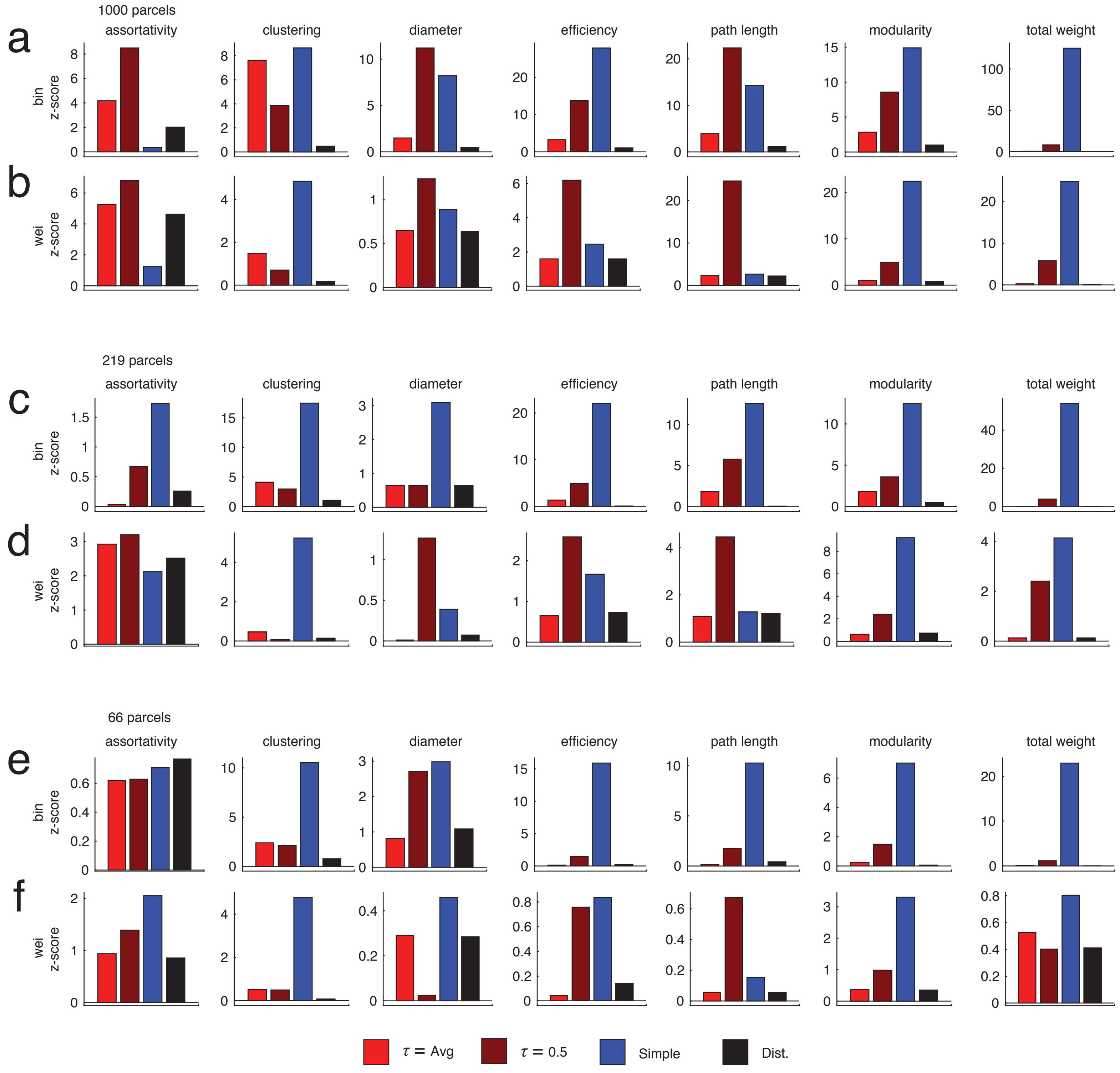

1000 parcels
a
b
219 parcels
c
d
66 parcels
e
f
assortativity
clustering
diameter
efficiency
path length
modularity
total weight
bin z-score
wei z-score
τ = Avg
τ = 0.5
Simple
Dist.

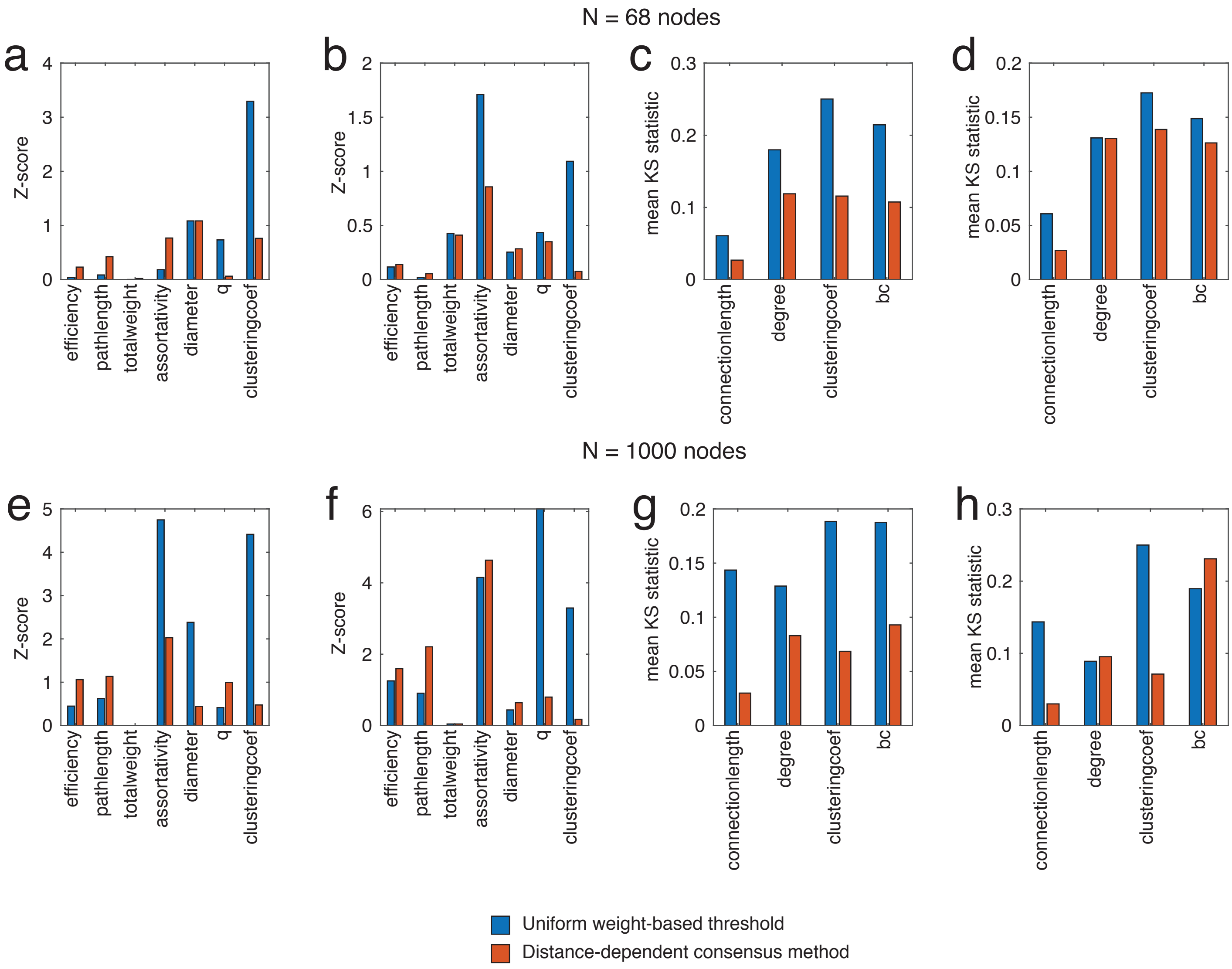

N = 68 nodes
a
b
c
d
Z-score
mean KS statistic
efficiency
pathlength
totalweight
assortativity
diameter
q
clusteringcoef
connectionlength
degree
bc
N = 1000 nodes
e
f
g
h
Uniform weight-based threshold
Distance-dependent consensus method
